# Structural basis for cooperative oxygen binding and bracelet-assisted assembly of Lumbricus terrestris hemoglobin

**DOI:** 10.1038/srep09494

**Published:** 2015-04-21

**Authors:** Wei-Ting Chen, Yu-Chuen Chen, Horng-Huei Liou, Chih-Yu Chao

**Affiliations:** 1 Department of Physics, National Taiwan University, Taipei 10617, Taiwan; 2 Graduate Institute of Applied Physics, National Taiwan University, Taipei 10617, Taiwan; 3 Biomedical & Molecular Imaging Center, National Taiwan University College of Medicine, Taipei 10051, Taiwan; 4 Division of Neurology, National Taiwan University Hospital, Taipei 10002, Taiwan

## Abstract

The iron-containing hemoglobins (Hbs) are essential proteins to serve as oxygen transporters in the blood. Among various kinds of Hbs, the earthworm Hbs are the champions in carrying oxygen due to not only their large size but also the unusually high cooperativity of ligand binding. However, the cooperative oxygen binding mechanisms are still mostly unknown. Here we report the cryo-electron microscopy structure of *Lumbricus terrestris* Hb in its native, oxygenated state at 9.1 Å resolution, showing remarkable differences from the carbon monoxide-binding X-ray structure. Our structural analysis first indicates that the cooperative ligand binding of *L. terrestris* Hb requires tertiary and quaternary transitions in the heme pocket and a global subunit movement facilitated by intra-ring and inter-ring contacts. Moreover, the additional sinusoidal bracelet provides the confirmation for the long-standing debate about the additional electron densities absent in the X-ray crystal structure.

We all need oxygen (O_2_), and so do most animals. The most widely distributed O_2_-carrying proteins in the animal kingdom are the iron-containing hemoglobins (Hbs). The remarkable cooperative binding ability of Hb and the allosteric communication between each binding site have fascinated scientists for more than a century. For tetrameric human Hb, the small shift of iron position in the heme groups results in large quaternary transitions between the unligated deoxy state and high affinity O_2_-ligated state^1^. In contrast, so far until now, no detectable ligand-binding induced structural changes have ever been observed in the giant hexagonal-bilayer earthworm Hb. The design of such huge macromolecular assembly has attracted scientists’ attentions for nearly two centuries. Some biological advantages may arise from the assemblage of such large and complex Hb. The giant size of earthworm Hb (3.6 million Daltons ~50 times the masses of human Hb) is required for being retained extracellularly within the vascular system. Besides, packing a lot of functional units into one particle can prevent too high viscosity of the blood while maximizing O_2_ transport. Due to their large size, extracellular nature, and resistance to oxidation, recent studies have shown that the Hb from the common earthworm *Lumbricus terrestris* could be a promising O_2_ carrier to be used in transfusion medicine^2^.

The earthworm *L. terrestris* Hb was the first protein to be crystallized in 1840^3^. It consists of 144 separate O_2_-binding globin subunits and 36 non-heme linker chains, where the linker chains stitch all the globin subunits together into one large assembly. Many structural studies by electron microscopy^4^,^5^,^6^ and X-ray crystallography^7^,^8^ have paved the way for a deeper understanding of this interesting molecular machine. The 3.5 Å resolution crystal structure of *L. terrestris* Hb reported by Royer and co-workers^7^ is a remarkable milestone. This structure provides the first atomic model for an entire megadalton respiratory protein, and reveals detailed hierarchical arrangement of 180 polypeptide chains. However, very little is known about the cooperative mechanism of O_2_ binding and about the structural transitions induced by different ligand binding. Crystallographers often use carbon monoxide (CO) as ligands to crystallize this large molecule and take it as a mimic for the oxygenated state because it is much more resistant to oxidation. However, no high resolution crystal structure has ever been solved for *L. terrestris* Hb in the real O_2_-liganded state.

The most intriguing functional characteristic of *L. terrestris* Hb is the unusually high cooperativity of O_2_ binding (Hill coefficient = 7.9, under conditions of maximum cooperativity)^9^, but no detectable structural change upon oxygenation was observed by resonance Raman spectra^10^ and small angle X-ray scattering^11^. The other interesting and recurring issue concerns the presence of a central subunit in the *L. terrestris* Hb. Ohtsuki and Crewe^12^ provided the first evidence for the central substructure in the *L. terrestris* Hb. The “bracelet model” proposed by Vinogradov *et al.*^13^ described the role of the central bracelet to act as linkers between globin subunits. The 3.5 Å resolution crystal structure revealed that the central subunits are formed by the N-terminal triple-stranded coiled coils of the linker chains. However, the smaller central cavity observed in some cryo-EM structures^6^,^14^,^15^ and the sinusoidal pillars observed in the 14.9 Å cryo-EM structure^4^ imply the plausible existence of additional central densities which are still absent in the X-ray structure.

In this paper, we report the 9.1 Å resolution cryo-EM structure of the entire *L. terrestris* Hb in the oxygenated form, which provides the first near-atomic resolution structure for this protein in its native state. By combining the 3.5 Å resolution crystal structure and the flexible fitting procedures, with our cryo-EM reconstruction, we construct a pseudo-atomic model for high-resolution description of the subunit arrangement in the oxygenated state. Comparison of the conformation of the *L. terrestris* Hb between two different functional states reveals tertiary and quaternary allostery in the heme pocket and an alteration of the overall size of this complex which provide clues to the cooperative mechanism. Moreover, the electron densities of additional sinusoidal bracelet are clearly visible in our cryo-EM data, and this discovery may account for a new assembly mechanism of the whole Hb complex.

## Results

### 9.1 Å resolution cryo-EM map of *L. terrestris* Hb

**Figure 1:**
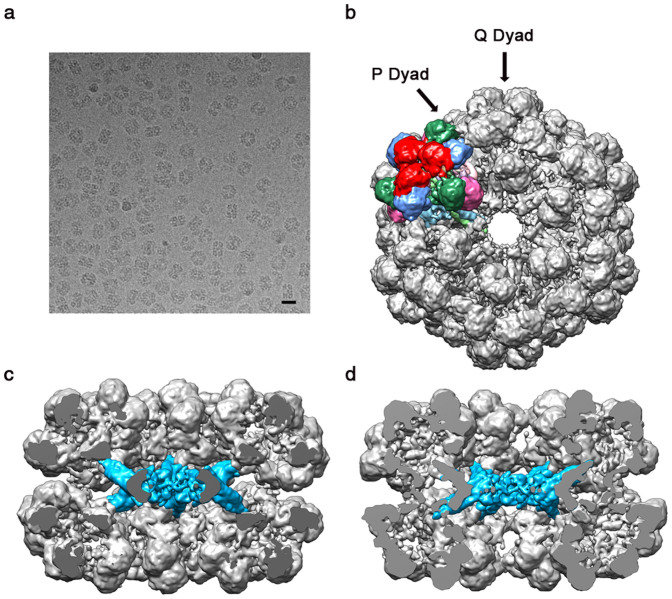
Final reconstruction of *Lumbricus terrestris* Hb at 9.1 Å resolution with one colored protomer. (a) A cryo-EM image of *Lumbricus terrestris* Hbs. Scale bar corresponds to 30 nm. (b) Top view along the molecular 6-fold axis. Individual subunits are shown in different colors, with a subunits in cornflower blue, b subunits in hot pink, c subunits in sea green, d subunits in red, linker chain L1 in light green, L2 in sky blue, and L3 in pink. Arrows point to the Q- and P-dyad axes. (c) The cut-away view along a Q-dyad axis. The central linker complex is shown in sky blue. (d) The cut-away view along a P-dyad axis. The cutting plane passes through the center of the molecule in (c) and (d).

To investigate the conformational change induced upon O_2_ binding, we carried out single particle cryo-EM analysis of *L. terrestris* Hb in its oxygenated state (Fig. 1a and 1b). No reference was made during the reconstruction process to avoid introducing model bias. The final 3D reconstruction is presented in Fig. 1, with one colored protomer. The map resolution was 9.1 Å according to the gold standard criterion (Supplementary Fig. 1). At 9.1 Å resolution, all the 144 globin subunits and 36 linker chains can be unambiguously assigned. The 36 non-heme linker chains formed a central core which acted as a scaffold covered by 144 O_2_ binding globins. Two unique dyad axes designated “Q” and “P” (Fig. 1b, 1c and 1d) oriented every 30° in the central plane reflected the D6 symmetry of the complex. The higher resolution of our cryo-EM map allows a clear visualization of each α-helix in the N-terminal triple-stranded coiled coils. This allowed unambiguous assignment of three linker chains, designated L1, L2, and L3 based on the known crystal structure. Interestingly, our cryo-EM map reveals additional electron densities in the N-terminal domain of the L1, L2 linker chains.

### Structural fitting

**Figure 2:**
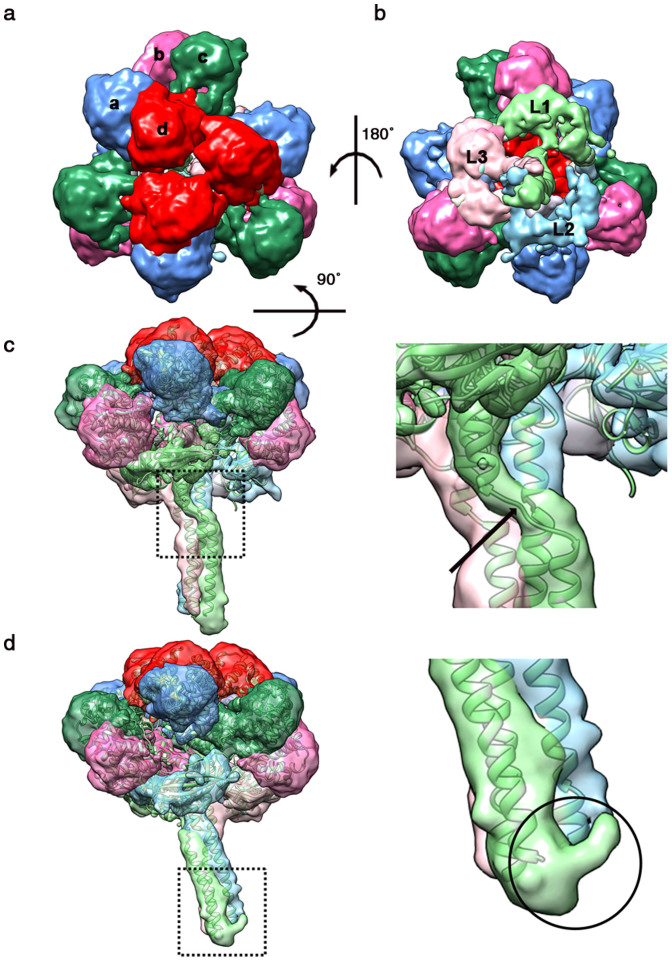
Cryo-EM density map and flexible fit of one protomer. (a) Top view along the pseudo three-fold axis shown colored as in Fig. 1. (b) Bottom view along the pseudo three-fold axis. The triple-stranded stalk can be seen from this view. (c) Side view of one protomer along the L1 linker chain. Arrow indicates the inter-helical loop of L1. (d) Side view along the L2 linker chain. Extra density is visible at the N terminus of L1 linker chain (black circle).

Since it is difficult to interpret the entire map, we extracted the protomer from the complex (Fig. 2a and 2b) and rigid-body docked the CO-binding form of *L. terrestris* Hb crystal structure into it. As the rigid body docking is performed using the entire protomer, the colied coil domain matches well, however, significant discrepancies are observed in the globin subunits and the β barrel domains. The overall cross-correlation coefficients is 0.70. The discrepancies between the O_2_-binding cryo-EM map and the CO-binding crystal structure suggested a conformational change induced by different ligand binding. To analyze the domain movements more accurately, we used flexible fitting protocol Flex-EM^16^ to dock the crystal structure into our EM map. This method maintains the connectivity between the domains and optimizes the position and orientation of each defined rigid body segment simultaneously^17^. The model for the protomer fits tightly into the density map (see Fig. 2c and 2d), except for some loop regions. The most remarkable discrepancies involve the extra densities in the N-terminal coiled coil domain. Compared to the crystal structure, the L1 linker chain is N-terminally extended by a unique tail domain (Fig. 2d), and finally forms a bracelet structure around the center of the assembled complex.

**Figure 3:**
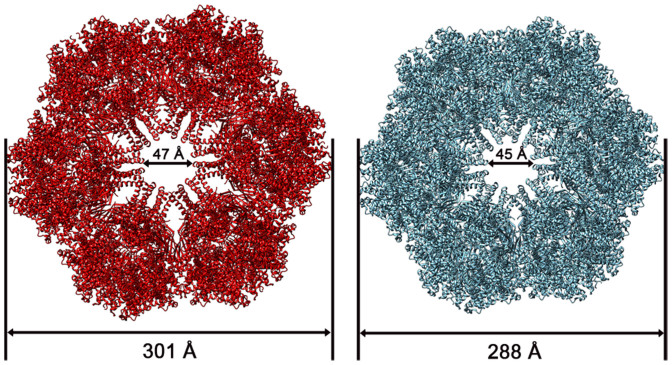
Comparison between the CO-bound state and O_2_-bound state of the *Lumbricus terrestris* Hb models. The CO and O_2_-bound states are shown in cyan and red, respectively.

After the protomer structure was flexibly docked into the density map, the whole map was symmetrically fitted while avoiding clashes between symmetrically placed molecules. The very high similarity between the density map and the flexible-fitting structure (cross-correlation coefficients ~0.94) allowed us to construct a pseudo-atomic model of the observed hexamer in the O_2_-binding state. With this approach, we were able to model the conformational change induced by O_2_ binding. The obtained O_2_-bound structure of *L. terrestris* Hb provides a different state to enable us to investigate in detail the effect of different ligand binding. After the flexible fitting procedure, the correlation coefficients increased to 0.94 (before flexible fitting ~0.7), which implies conformational difference between CO and O_2_ binding states. The average root-mean-square deviation (RMSD) of the overall complex between the fitted model and the CO binding X-ray structure was 6.6 Å. Figure 3 shows the comparison of the *L. terrestris* Hb models in the O_2_-bound and CO-bound states. Upon binding of the O_2_ molecules, each of the protomers moves outward along the quasi 3-fold axis, causing a radial expansion of the hexagonal bilayer complex. The fitted pseudo-atomic model has a diameter of 301 Å and a height of 193 Å while the CO-bound state has a 288 Å diameter and 186 Å height. On the inside, the cavity of the hexamer after O_2_ binding is 47 Å in diameter and remains almost unchanged. Even though the inside cavity diameter of the CO-bound crystal structure still matches that of the O_2_-bound complex, the outside diameter is 13 Å narrower in CO-binding state.

Each of 12 protomers is composed of a globin dodecamer which binds to the head of a linker heterotrimer. The twelve globin subunits (abcd)_3_ in each protomer appear to group into six peanut-like subunit pairs (a–d, b–c) that create a pseudo three-fold symmetry (Fig. 2a), whereby each protomer is considered to be a trimer of tetramers. The L1, L2, and L3 linker chains are held together as a trimer by disulfide bonds and strong hydrophobic interactions^7^. Each linker chain is comprised of a long N-terminal α helix, a low-density lipoprotein receptor (LDLR)-like domain^18^, and a typical eight-stranded β barrel domain^7^. A nonhelical region divides the N-terminal α helix into a long coiled coil and a shorter one near the β barrel domain. It was also known that linker chain L1 exhibits the longest inter-helical segment which introduces a break in the crystal structure^7^. Thus, we refined the inter-helical loop by generating additional possible conformations^19^ which fit our density map more accurately (Fig. 2c).

### Central linker complex

**Figure 4:**
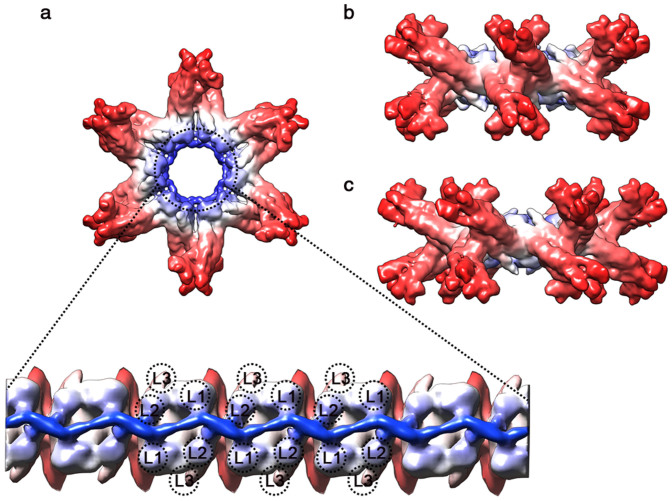
The cryo-EM electron density map of the central linker complex. (a) Top view along the molecular 6-fold axis. The density map is colored by radius with a red-to-blue spectrum. The electron densities found inside the black dashed circle unambiguously indicate the existence of the central bracelet structure which appears as a continuous sinusoid in the enlarged unwrapped planar map. (b) The complex viewed along a Q-dyad axis. (c) The complex viewed along a P-dyad axis.

The linker trimer has a long stalk at the center of *L. terrestris* Hb, formed from the triple-stranded coiled coil. The coiled coil domains provide the primary contacts between one-twelfth protomers that form the overall hexagonal bilayer structure. At this resolution, the triple-stranded coiled coils were clearly evident. In order to demonstrate the arrangement of the 36 linker chains at the core of the complex, we masked out the globin subunits and a portion of the linker chains (Fig. 4a). Then we found that the two hexagonal rings are staggered, so that the stalks appear curved between the top and bottom rings when viewed from the Q dyad (Fig. 4b). Each triple-stranded stalk projected from outside toward the main plane of the central linker complex (Fig. 4c) are arranged in an alternating manner. The angle between the top and bottom stalks is approximately 45° (Fig. 4b). Interestingly, our map also reveals additional densities in the N termini of the linker chains, which make the central cavity much smaller than that of the X-ray structure. The extra density forms a bracelet structure which connects all the coiled coil domains of L1 and L2 linker chains in the assembled complex (Fig. 4a). These features are totally absent in the X-ray structure for unknown reasons^4^. The interactions formed by the additional electron densities observed in our cryo-EM map might be the potential forces making the complex more compact and stable.

The interdigitation between the extra density and the L1, L2 coiled coils can be appreciated from the unwrapped planar map (Fig. 4a). As shown in the unrolled central linker complex, the extra density appears as a continuous sinusoid which was presented as six sinusoidal pillars in the 14.9 Å cryo-EM structure^4^. To further investigate the changes occurred in the coiled coil domain, we compare the extracted coiled coil structures from the O_2_-bound and CO-bound states. By superimposing their structures, the local conformational changes within the subunit can be examined. One apparent structural change upon oxygenation is in the short coiled coil of L1 linker chain (see Supplementary Fig. 2b). The short coiled coil of L1 tilts toward the central plane with the hinge point near the inter-helical loop. The conformations of L2 and the long coiled coil of L1 which were connected by the sinusoidal bracelet are almost unchanged.

### Interactions between protomers

**Figure 5:**
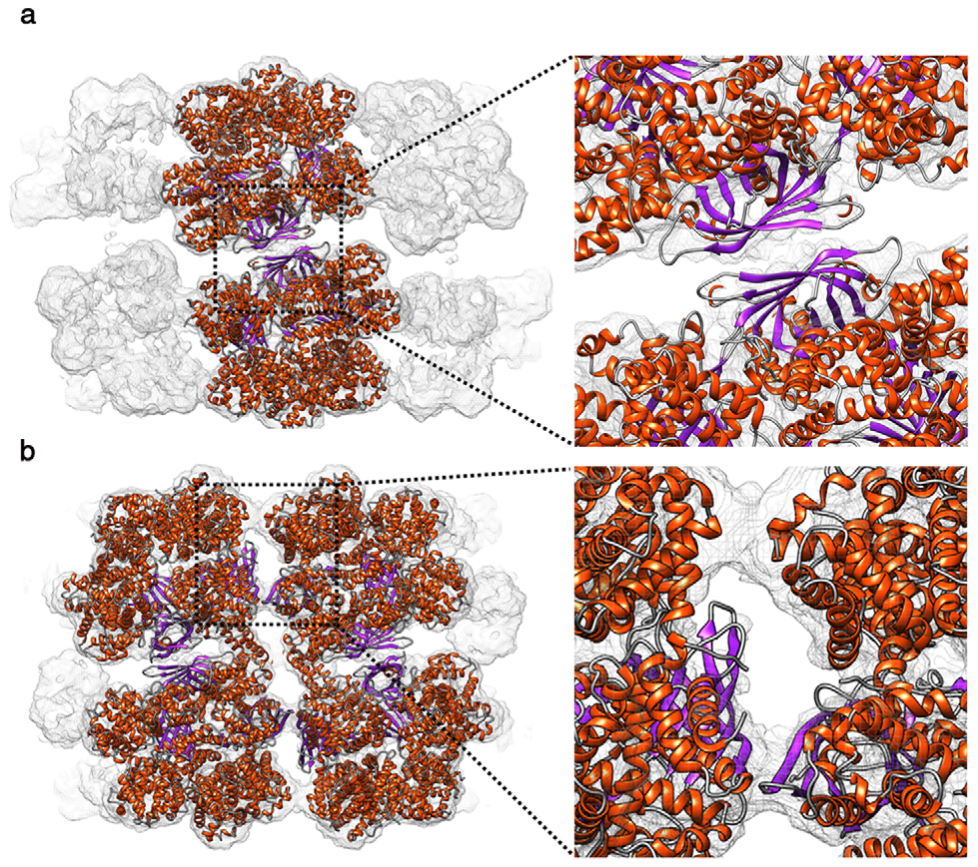
Contacts between protomers. (a) Inter-ring contacts along the Q-dyad. The model is colored by secondary structure with helix in orange red and strand in purple. (b) Intra-ring contacts between lateral protomers.

Except for the primary contacts made by the coiled coil domains, two distinct inter-ring and intra-ring contacts are formed between neighboring protomers. The most extensive inter-ring contacts occur along the Q-dyad. As shown in Fig. 5a, the β barrel domains of L1 linker chain from two protomers pack together at the Q-dyad. The intra-ring contacts include interactions between globin dodecamers and the β barrel domains of L2 and L3 linker chains (Fig. 5b). The two hexagonal layers of *L. terrestris* Hb are partially staggered such that the architecture is more compact than the type II Hb^5^. Despite the closer manner of two hexagonal layers, the globin subunits actually do not have direct inter-ring interactions. It’s the β barrel domain of L1 linker chain providing the only inter-ring contacts, which could have an important role coordinating inter-ring allostery. Supplementary Fig. 2a shows the Q-dyad contact of L1 linker chains from different ligand-bound states. On going from CO to O_2_-bound Hb, the β barrel domains and the LDLR-like domain of both rings are translated outward from the center of the molecule (Supplementary Movie 1). The translation of the Q-dyad contact is associated with the tilting of the short helix of the L1 coiled coil domain. The inter-helical loop of L1 linker chain seems to act as a flexible hinge to allow relative motion while maintaining the rigidity of the central linker complex.

### Conformational change in the heme pocket

**Figure 6:**
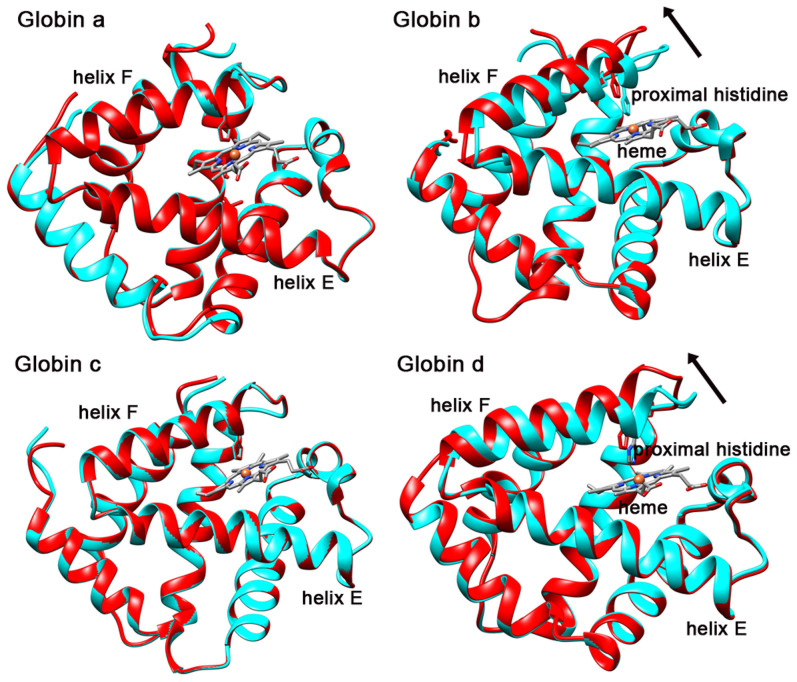
Comparison of the heme pocket of each globin subunit in CO-bound state (cyan) and O_2_-bound state (red). The arrow indicates the tilting direction of helix F of globin b and globin d. The proximal histidine is away from the heme group in O_2_-bound state.

The extracellular *L. terrestris* Hb comprises four unique heme-containing subunits, a, b, c, and d in equal proportions. The four globin subunits exhibit the standard myoglobin fold, with seven α helices designated A through H (no D-helix)^8^. The heme group was contained in the E and F helices. Assembly of the globin dodecamer was dictated by four unique interfacial contacts, including two distinct EF dimer interfaces, one intra-tetramer interface and one inter-tetramer interface^8^. EF dimer pairing has been observed in all cooperative invertebrate hemoglobins to date^20^, in which an extensive dimeric interface forms from contacts involving the heme containing E and F helices. In order to find the tertiary changes occurred in the heme pocket, we compared each globin subunit from the O_2_-bound and CO-bound states. Their structures were superimposed for comparison and analyses (Fig. 6). In this way, we can examine the local tertiary changes within the subunit, which is independent of the quaternary movement. The RMSD over each entire globin domain is small (less than 1 Å), which implies most of the residues match well in the CO and O_2_ binding states. However, the RMSD increases from the beginning of the F helix and has a local maximum ~3.1 Å near the proximal histidine. In our results, one remarkable tertiary conformational change upon O_2_ binding is in the helix F which contains the proximal histidine for interaction with the heme group (Supplementary Movie 2). By interacting with the heme iron atom, the helix F tilts upward from the heme plane upon O_2_ binding. Since we can not determine the location of the heme group by the cryo-EM map at this resolution, the heme group is positioned relative to the CO-bound crystal structure. Interestingly, the tilt of the helix F only occurs in the subunit b and subunit d. In contrast to subunit b and subunit d, subunit a and subunit c show no obvious tertiary change between two states.

## Discussion

The focus of our research was to investigate the structural transitions induced by different ligand binding to the giant hexagonal-bilayer Hb of *L. terrestris*. The combination of cryo-EM structure and the flexible fitting technique provides a good means for studying the tertiary and quaternary structure in the specific functional state. Earlier resonance Raman spectra^10^ and small angle x-ray scattering studies^11^ have previously pointed that the giant *L. terrestris* Hb lacked ligand-binding induced tertiary and quaternary changes. On the contrary, our results indicate that the ligand-induced structural changes of *L. terrestris* Hb contain both tertiary and quaternary allostery which may account for the unusually high cooperativity of O_2_ binding. Since the resonance Raman observations reflect an average behavior of all the heme pockets, it can not be ruled out that a small number of heme environments could undergo ligand-induced conformational change. Indeed, our results show that only the heme pocket in globin b and globin d undergoes tertiary changes. The distinctive feature in these two subunits could be due to the tryptophan at position B10 that reaches into the binding heme pocket, making van der Waals contacts with the gas ligand^8^. It is reasonable to attribute the tertiary changes to the interaction between the tryptophan and the gas ligand. The structures shown indicate that the proximal histidine becomes detached from the heme iron. The proximal histidine in the F helix^21^ is a strictly conserved residue in the hemoglobin which helps to coordinate the heme iron. Actually, it is the only point of direct interaction between the heme and protein^8^. The pioneering studies of Max Perutz highlighted roles of the proximal histidine in regulating the oxygen affinity by allosteric effect^21^,^22^. According to Perutz’s model, the proximal histidine comes closer to the heme plane in oxy than in deoxyhemoglobin^23^,^24^. Geometric analysis and Monte Carlo simulation indicate that the oxygenated conformation lies along the pathway between deoxy and CO-binding states^25^,^26^. Moreover, analysis of Hb structures in the Protein Data Bank also reveals the reaction pathway is T (deoxy) -R (O_2_ binding) -R2 (CO binding)^27^,^28^. Therefore, such a movement of the F-helix of our data is what would be expected as a difference between oxygenated and CO liganded hemoglobins. The allosteric signal is transmitted through the heme iron to the proximal histidine, causing the tilting of helix F in globin b and d away from the heme plane. The structural transitions found here can explain the unusually high cooperativity of O_2_ binding in the *L. terrestris* Hb which has long been an unsolved mystery in the past. Here our results show that the overall cooperativity of a hexagonal bilayer complex is caused by the plurality of subunit-subunit interactions in this assemblage, which is quite different from those of the vertebrate and other invertebrate Hbs. The earthworm Hb requires assembly of multiple copies of four distinct globin subunits while the higher vertebrates invariably display the α_2_β_2_ tetrameric form^20^. The retention of only partial cooperativity by incomplete assembly^29^,^30^ implies that the full cooperativity relies on the presence of multiple subunit-subunit interactions in the hierarchy of the assembly. Besides the tertiary and quaternary allostery in the heme pocket, there is an alteration of the overall size of the complex. Upon oxygen binding, the allosteric signal is transmitted through the heme iron to the proximal histidine, causing the tilting of helix F. In the next step, the allosteric signal is transmitted to the linker via the most extensive contacts that involve the β barrel and LDLR-like domain^7^. One interesting interaction occurs between globin b and LDLR-like domain. The Arg B11 from globin b subunit reaches into the LDLR-like domain to make very favorable interactions with Asp 88^7^ (L1 numbering). The involvement of residue B11, next to B10 in the binding pocket, suggests that this contact could have implications for the allosteric regulation. Finally, the short helix of L1 tilts outward around the flexible hinge (inter-helical loop) (Supplementary Fig. 2b), and the β barrel domain was pushed outward causing the radial expansion of the whole complex (Supplementary Movie 1). The oxygenation causes a radial expansion of the hexagonal-bilayer complex while maintaining the rigidity of the central linker complex. Considering the error margins of fitting (~4 Å), there is still ~9 Å radial expansion of the whole hemoglobin complex between our cryo-EM data (301 Å) in the O_2_-bound state and the known crystal structure (288 Å) in the CO-bound state^31^. This global subunit movement is found to be accomplished by the allosteric communications between intra-ring and inter-ring contacts. The intra-ring interactions primarily involve main chain hydrogen bonding between β barrel domains of L2 and L3^7^, while the inter-ring contacts involve the extensive packing of the β barrel domains of L1 as seen in Fig. 5a. This emphasizes the critical role of linker chains in the allosteric behavior exhibited by this macromolecule. One interesting feature is the longest inter-helical segment of L1, which serves as a flexible hinge region (Supplementary Fig. 2b) and allows structural transitions of the short coiled coil relative to the longer one. The allosteric role of linker chains observed in our results provides a reasonable explanation for the smaller cooperativity of dodecamer^11^ and Riftia Hb^32^ without linker chains.

Another unanswered issue in the annelid Hb literatures concerns the presence of the additional densities in the central linker complex. The so-called “bracelet model”^13^ had been proposed to explain the assembly of the quaternary structure by the central bracelet. Some of the early publications on *L. terrestris* Hb^6^,^12^,^15^,^33^ provided some evidence of visible density in the center of the Hb macromolecule. The 3.5 Å resolution crystal structure revealed the interdigitation of 12 triple-stranded coiled-coils near the center of the complex. The smaller central cavity and the sinusoidal pillars observed in the 14.9 Å cryo-EM structure^4^ gave a further support of the additional densities which were absent in the crystal structure for unknown reasons. These findings, however, do not close the discussions because no detailed and complete structural evidence was provided so far. Our 9.1 Å resolution cryo-EM structure clearly reveals the details of the additional sinusoidal bracelet (Supplementary Fig. 3) that further enhance the completeness of the structural interdigitations between 12 triple-stranded coiled-coils. Moreover, the two smallest RMSD values in the L2 (2.25 Å) and L1 (2.52 Å) linker chains between the fitted model and the X-ray structure strongly suggest the structural role that the extra densities played to reinforce the strength of the central linker complex. The absolute requirement for the L1 linker chain for the Hb assemblage and the interchangeability between L1 and L2^33^ also support the interdigitations between the extra densities (Fig. 4a) and the L1 and L2 linker chains.

This study yields important implications for the allosteric mechanisms of the giant hexagonal bilayer Hb based on structural comparison and analysis. The tertiary and quaternary allostery and the allosteric communications facilitated by intra-ring and inter-ring contacts underlie the requirement for distinct hemoglobin sequences and the non-heme linker chains. The flexibility of the β barrel domain and the rigidity of the central linker complex strengthened by the extra bracelet clarify the dual functions of the linker chains. Besides, the radial expansion in the oxygenated state of *L. terrestris* Hb complex was confirmed and analyzed by cryo-EM and flexible fitting technique, which are good means to register protein dynamics in native environment. It remains to be seen what constituents could account for the additional sinusoidal densities. One possibility may be that the additional densities could correspond to the N-terminal residues of L1 and L2 linkers which were not observed in the crystal structure. Structural analysis at higher resolution will be required to elucidate how the allosteric units drive global movement, and how the structural transitions alter the O_2_ binding affinity. The determination of the corresponding structures at specific functional states is a matter of vital importance.

## Methods

### Hemoglobin purification

The common earthworm *L. terrestris* was purchased from Carolina Biological Supply Co. (Burlington, NC). Live earthworms were washed in distilled water to remove dirt and mucus, dried off, and cut near the seventh body segment. The blood was collected via capillary tubes into a centrifugal tube containing 0.1 M Tris-HCl buffer, pH 7.0, 1 mM EDTA. The collected blood was centrifuged at 20,000 × *g* for 0.5 hr at 4 degrees Celsius to remove any cell debris and particulate matter. The supernatant was then centrifuged at 150,000 × *g* for 2 hr at 4 degrees Celsius. The red pellet was dissolved in the Tris-HCl buffer and centrifuged at 150,000 × *g* again. The final pellet was dissolved in 0.1 M Tris-HC1 buffer, pH 7.0, 1 mM EDTA, 2 mM phenylmethanesulfonyl fluoride (PMSF)^34^.

### Sample preparation for electron microscopy analysis

For cryo-EM, the Hb sample was dialyzed against a 50 mM Tris-HCl buffer, pH 7.2, 10 mM CaCl_2_, 10 mM MgCl_2_ to a concentration of 2 mg/ml. Approximately 3.5 µl of the sample dilution was applied onto holey carbon side of each freshly glow-discharged Quantifoil copper grid (R2/2, Quantifoil Micro Tools GmbH, Jena, Germany). After blotting at room temperature for 3.5 s at 100% humidity in the Vitrobot (FEI, Netherlands), the grid was rapidly plunged into liquid ethane cooled by liquid nitrogen.

### Electron microscopy

Grids of frozen hydrated samples were transferred into the electron microscope by using the Gatan 626 cryo-transfer system (Gatan, USA). The cryo specimens were imaged in a Tecnai F20 (FEI, Netherlands) electron microscope operated at an acceleration voltage of 200 kV. Low dose images were recorded at a nominal magnification of 80,000 using a Gatan UltraScan 4000 4 k × 4 k CCD camera (Gatan, USA) by an automated data acquisition system, Leginon^35^.

### Image processing and 3D reconstruction

The quality of the CCD images were determined by their power spectra. Micrographs with noticeable drift, charging or astigmatism were discarded. The defocus and astigmatism parameters of each micrograph were determined using the program CTFFIND3^36^, and the contrast transfer function (CTF) correction was done with the module CTF2D-FLIP from IMAGIC^37^. All the particles without contaminants and aggregates were semi-automatically selected using the EMAN2 tool e2boxer^38^. Approximately 6,000 particles were selected from 250 micrographs. Particles were then extracted into boxes of 400 × 400 pixels, band-pass filtered between 200 and 3 Å, and normalized to a constant mean and standard deviation using IMAGIC. To increase the speed of the alignment process, the pretreated particles were first coarsened to 200 × 200 pixels and band-pass filtered between 200 and 10 Å. Particles were translationally centered to the total sum of the dataset and classified by multivariate statistical analysis (MSA) in IMAGIC with approximately 7 images per class. Ten best class averages with characteristic views were selected as references for multi-reference alignment (MRA). After three rounds of MSA/MRA cycle, an initial map was calculated from class averages by angular reconstitution with D6 symmetry imposed. Reprojections of the initial model were used as an anchor set for angular refinement, after angular reconstitution, a new 3D model was calculated. The angular refinement process was then repeated iteratively until the euler angles stabilize. Subsequently, particle orientations were refined by several cycles of MRA, MSA and angular reconstitution until a reasonable convergence was achieved. Finally, the alignment parameters were needed to be refined on the un-coarsened dataset, including the high frequency terms, by the same iterative cycle. In our 3D reconstruction data reported here, the reconstructed map was further refined by twelve cycles of projection matching procedure implemented in IMAGIC with a progressively decreased angular increment (with a final value of 1° only). After each competitive projection matching, particles were classified into different defocus groups and 75% of the best particles of each group (according to their cross-correlation coefficients) were selected for 3D reconstruction. The resolution of the structure was assessed according to the Fourier shell correlation (FSC) of two maps calculated separately from two halves of the dataset.

### Structure analysis and EM density fitting

The 3D volume was analyzed, segmented and visualized by UCSF Chimera^39^. The CO-binding crystal structure (PDB code: 2GTL) protomer was first rigid-body fitted into the segmented cryo-EM map by Chimera. Then the conformation refinement was performed by the simulated annealing molecular dynamics optimization protocol embedded in the Flex-EM software^16^. During the flexible fitting process, the secondary structural elements detected by RIBFIND^17^ were displaced in the direction which maximized their cross-correlations with the cryo-EM density map. After the protomer structure was flexibly fitted into the cryo-EM density map, the whole map was symmetrically fitted while avoiding clashes between neighboring subunits by UCSF Chimera.

## Additional information

Accession codes: The electron density map and fitted atomic models have been deposited in the EBI, www.ebi.ac.uk (accession no. EMD-2627), and Protein Data Bank, www.pdb.org (PDB ID code 4V93).

## Supplementary Material

Supplementary Information Supplementary information

Supplementary Information Supplementary Movie 1

Supplementary Information Supplementary Movie 2

## References

[b1] Ackers G. K. & Holt J. M. Asymmetric cooperativity in a symmetric tetramer: human hemoglobin. J. Biol. Chem. 281, 11441–11443 (2006). 16423822 10.1074/jbc.R500019200

[b2] Elmer J., Palmer A. F. & Cabrales P. Oxygen delivery during extreme anemia with ultra-pure earthworm hemoglobin. Life Sci. 91, 852–859 (2012). 22982347 10.1016/j.lfs.2012.08.036 PMC3511863

[b3] Reichert E. T. & Brown A. P. The crystallography of hemoglobins. Exp. Biol. Med. 5, 66–68 (1908).

[b4] Mouche F., Boisset N. & Penczek P. A. Lumbricus terrestris hemoglobin–the architecture of linker chains and structural variation of the central toroid. J. Struct. Biol. 133, 176–192 (2001). 11472089 10.1006/jsbi.2001.4362

[b5] Jouan L., Taveau J.-C., Marco S., Lallier F. H. & Lamy J. N. Occurrence of two architectural types of hexagonal bilayer hemoglobin in annelids: comparison of 3D reconstruction volumes of Arenicola marina and Lumbricus terrestris hemoglobins. J. Mol. Biol. 305, 757–771 (2001). 11162090 10.1006/jmbi.2000.4344

[b6] Schatz M., Orlova E. V., Dube P., Jger J. & van Heel M. Structure of Lumbricus terrestris hemoglobin at 30 Å resolution determined using angular reconstitution. J. Struct. Biol. 114, 28–40 (1995). 7772416 10.1006/jsbi.1995.1003

[b7] Royer W. E. Jr, Sharma H., Strand K., Knapp J. E. & Bhyravbhatla B. Lumbricus erythrocruorin at 3.5 Å resolution: architecture of a megadalton respiratory complex. Structure 14, 1167–1177 (2006). 16843898 10.1016/j.str.2006.05.011

[b8] Strand K., Knapp J. E., Bhyravbhatla B. & Royer W. E. Jr Crystal structure of the hemoglobin dodecamer from Lumbricus erythrocruorin: allosteric core of giant annelid respiratory complexes. J. Mol. Biol. 344, 119–134 (2004). 15504406 10.1016/j.jmb.2004.08.094

[b9] Fushitani K., Imai K. & Riggs A. F. Oxygenation properties of hemoglobin from the earthworm, Lumbricus terrestris. Effects of pH, salts, and temperature. J. Biol. Chem. 261, 8414–8423 (1986). 3722158

[b10] Vidugiris G. J., Harrington J. P., Friedman J. M. & Hirsch R. E. Absence of ligand binding-induced tertiary changes in the multimeric earthworm Lumbricus terrestris hemoglobin. A resonance Raman study. J. Biol. Chem. 268, 26190–26192 (1993). 8253738

[b11] Krebs A. *et al.* Molecular shape, dissociation, and oxygen binding of the dodecamer subunit of Lumbricus terrestris hemoglobin. J. Biol. Chem. 271, 18695–18704 (1996). 8702524 10.1074/jbc.271.31.18695

[b12] Ohtsuki M. & Crewe A. V. Evidence for a central substructure in a Lumbricus terrestris hemoglobin obtained with STEM low-dose and digital processing techniques. J. Ultrastruct. Res. 83, 312–318 (1983). 6876253 10.1016/s0022-5320(83)90138-7

[b13] Vinogradov S. N., Lugo S. D., Mainwaring M. G., Kapp O. H. & Crewe A. V. Bracelet protein: a quaternary structure proposed for the giant extracellular hemoglobin of Lumbricus terrestris. Proc. Natl. Acad. Sci. USA 83, 8034–8038 (1986). 3464939 10.1073/pnas.83.21.8034 PMC386861

[b14] Lamy J. N. *et al.* Giant hexagonal bilayer hemoglobins. Chem. Rev. 96, 3113–3124 (1996). 11848854 10.1021/cr9600058

[b15] de Haas F. *et al.* Three-dimensional reconstruction of native and reassembled Lumbricus terrestris extracellular hemoglobin. Localization of the monomeric globin chains. Biochemistry 36, 7330–7338 (1997). 9200681 10.1021/bi970131l

[b16] Topf M. *et al.* Protein structure fitting and refinement guided by cryo-EM density. Structure 16, 295–307 (2008). 18275820 10.1016/j.str.2007.11.016 PMC2409374

[b17] Pandurangan A. P. & Topf M. Finding rigid bodies in protein structures: application to flexible fitting into cryoEM maps. J. Struct. Biol. 177, 520–531 (2012). 22079400 10.1016/j.jsb.2011.10.011

[b18] Suzuki T. & Riggs A. F. Linker chain L1 of earthworm hemoglobin. Structure of gene and protein: homology with low density lipoprotein receptor. J. Biol. Chem. 268, 13548–13555 (1993). 8514788

[b19] Sali A. & Blundell T. L. Comparative protein modelling by satisfaction of spatial restraints. J. Mol. Biol. 234, 779–815 (1993). 8254673 10.1006/jmbi.1993.1626

[b20] Royer W. E. Jr, Zhu H., Gorr T. A., Flores J. F. & Knapp J. E. Allosteric hemoglobin assembly: diversity and similarity. J. Biol. Chem. 280, 27477–27480 (2005). 15932877 10.1074/jbc.R500006200

[b21] Royer W. E. Jr, Knapp J. E., Strand K. & Heaslet H. A. Cooperative hemoglobins: conserved fold, diverse quaternary assemblies and allosteric mechanisms. Trends in Biochemical Sciences 26, 297–304 (2001). 11343922 10.1016/s0968-0004(01)01811-4

[b22] Mouawad L., Perahia D., Robert C. H. & Guilbert C. New Insights into the Allosteric Mechanism of Human Hemoglobin from Molecular Dynamics Simulations. Biophysical journal 82, 3224–3245 (2002). 12023247 10.1016/S0006-3495(02)75665-8 PMC1302112

[b23] Perutz M. F. Stereochemistry of cooperative effects in haemoglobin: haem-haem interaction and the problem of allostery. Nature 228, 726–734 (1970). 5528785 10.1038/228726a0

[b24] Perutz M. F. Mechanisms regulating the reactions of human hemoglobin with oxygen and carbon monoxide. Annu. Rev. Physiol. 52, 1–25 (1990). 2184753 10.1146/annurev.ph.52.030190.000245

[b25] Lukin J. A. & Ho C. The structurefunction relationship of hemoglobin in solution at atomic resolution. Chem. Rev. 104, 1219–1230 (2004). 15008621 10.1021/cr940325w

[b26] Kantarci-Carsibasi N., Haliloglu T. & Doruker P. Conformational Transition Pathways Explored by Monte Carlo Simulation Integrated with Collective Modes. Biophys. J. 95, 5862–5873 (2008). 18676657 10.1529/biophysj.107.128447 PMC2599820

[b27] Srinivasan R. & Rose G. D. The T-to-R transformation in hemoglobin: a reevaluation. Proc. Natl. Acad. Sci. USA 91, 11113–11117 (1994). 7972019 10.1073/pnas.91.23.11113 PMC45177

[b28] Ren Z. Reaction Trajectory Revealed by a Joint Analysis of Protein Data Bank. PLoS One 8, e77141 (2013). 24244274 10.1371/journal.pone.0077141 PMC3823880

[b29] Vinogradov S. N. *et al.* A dodecamer of globin chains is the principal functional subunit of the extracellular hemoglobin of Lumbricus terrestris. J. Biol. Chem. 266, 13091–13096 (1991). 2071593

[b30] Fushitani K. & Riggs A. F. The extracellular hemoglobin of the earthworm, Lumbricus terrestris. Oxygenation properties of isolated chains, trimer, and a reassociated product. J. Biol. Chem. 266, 10275–10281 (1991). 2037579

[b31] Royer W. E. Jr, Strand K., van Heel M. & Hendrickson W. A. Structural hierarchy in erythrocruorin, the giant respiratory assemblage of annelids. Proc Natl Acad Sci USA 97, 7107–7111 (2000). 10860978 10.1073/pnas.97.13.7107 PMC16507

[b32] Arp A. J., Doyle M. L., Di Cera E. & Gill S. J. Oxygenation properties of the two co-occurring hemoglobins of the tube worm Riftia pachyptila. Resp. Physiol. 80, 323–334 (1990). 10.1016/0034-5687(90)90092-d 2218103

[b33] Lamy J., Kuchumov A., Taveau J.-C., Vinogradov S. N. & Lamy J. N. Reassembly of Lumbricus terrestris hemoglobin: a study by matrix-assisted laser desorption/ionization mass spectrometry and 3D reconstruction from frozen-hydrated specimens. J. Mol. Biol. 298, 633–647 (2000). 10788326 10.1006/jmbi.2000.3689

[b34] Vinogradov S. N. & Sharma P. K. Preparation and characterization of invertebrate globin complexes. Methods Enzymol. 231, 112–124 (1994). 8041246 10.1016/0076-6879(94)31010-6

[b35] Suloway C. *et al.* Automated molecular microscopy: the new Leginon system. J. Struct. Biol. 151, 41–60 (2005). 15890530 10.1016/j.jsb.2005.03.010

[b36] Mindell J. A. & Grigorieff N. Accurate determination of local defocus and specimen tilt in electron microscopy. J. Struct. Biol. 142, 334–347 (2003). 12781660 10.1016/s1047-8477(03)00069-8

[b37] van Heel M., Harauz G., Orlova E. V., Schmidt R. & Schatz M. A new generation of the IMAGIC image processing system. J. Struct. Biol. 116, 17–24 (1996). 8742718 10.1006/jsbi.1996.0004

[b38] Tang G. *et al.* EMAN2: an extensible image processing suite for electron microscopy. J. Struct. Biol. 157, 38–46 (2007). 16859925 10.1016/j.jsb.2006.05.009

[b39] Goddard T. D., Huang C. C. & Ferrin T. E. Visualizing density maps with UCSF Chimera. J. Struct. Biol. 157, 281–287 (2007). 16963278 10.1016/j.jsb.2006.06.010

