# Supplementary material for: Structural basis for cooperative oxygen binding and bracelet-assisted assembly of Lumbricus terrestris hemoglobin
Source: Sci Rep. 2015 Apr 21;5:9494. doi: 10.1038/srep09494 (PMC5383013; doi:10.1038/srep09494)
Supplement: Supplementary Information [file srep09494-s1.doc]

Structural basis for cooperative oxygen binding and bracelet-assisted assembly of Lumbricus terrestris hemoglobin

Wei-Ting Chen^1,3^, Yu-Chuen Chen^3^, Horng-Huei Liou^4^ & Chih-Yu Chao^1,2,3,*^

^1^Department of Physics, National Taiwan University, Taipei 10617, Taiwan

^2^Graduate Institute of Applied Physics, National Taiwan University, Taipei 10617, Taiwan

^3^Biomedical & Molecular Imaging Center, National Taiwan University College of Medicine, Taipei 10051, Taiwan

^4^Division of Neurology, National Taiwan University Hospital, Taipei 10002, Taiwan

*Correspondence: [cychao@ntu.edu.tw](mailto:cychao@ntu.edu.tw)





**Supplementary Figure 1****.** **Fourier shell correlation resolution curve.** Resolution determination by Fourier shell correlation of two maps calculated separately from two halves of the dataset. The gold standard FSC curve (gray) was plotted along with the conventional curve. The estimated resolution of *L. terrestris* Hb was 9.1 Å according to the gold standard criterion.


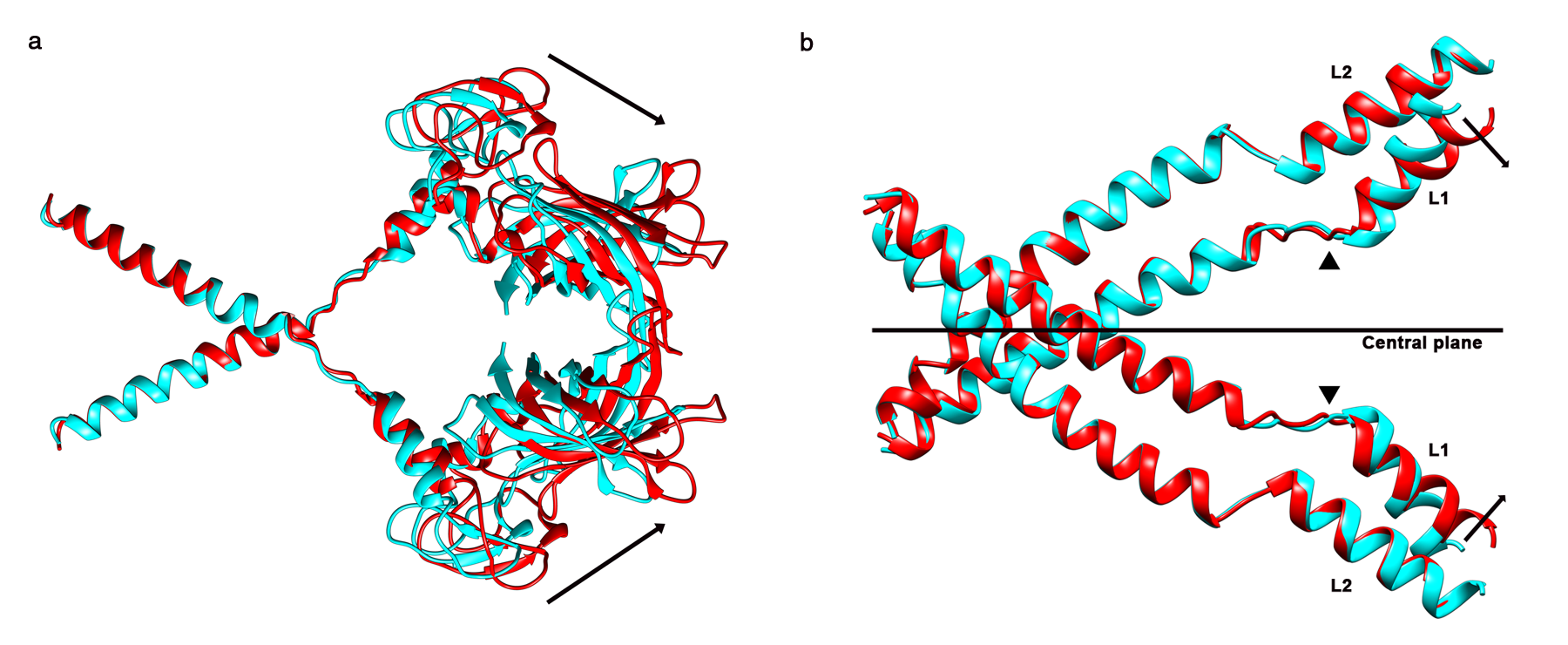


**Supplementary Figure 2.** **Comparison of the Q-dyad contact and coiled coil domain in CO-bound state (cyan) and O_2_-bound state (red).** (**a**) Comparison of the Q-dyad contact of L1 linker chains. The arrow indicates the transition direction of the β barrel domains and the LDLR-like domains going from CO to O_2_-bound state. (**b**) Comparison of the coiled coil domain. The tilt of the short coiled coil of L1 around the hinge point (marked by a triangle) near the inter-helical loop is indicated by an arrow.


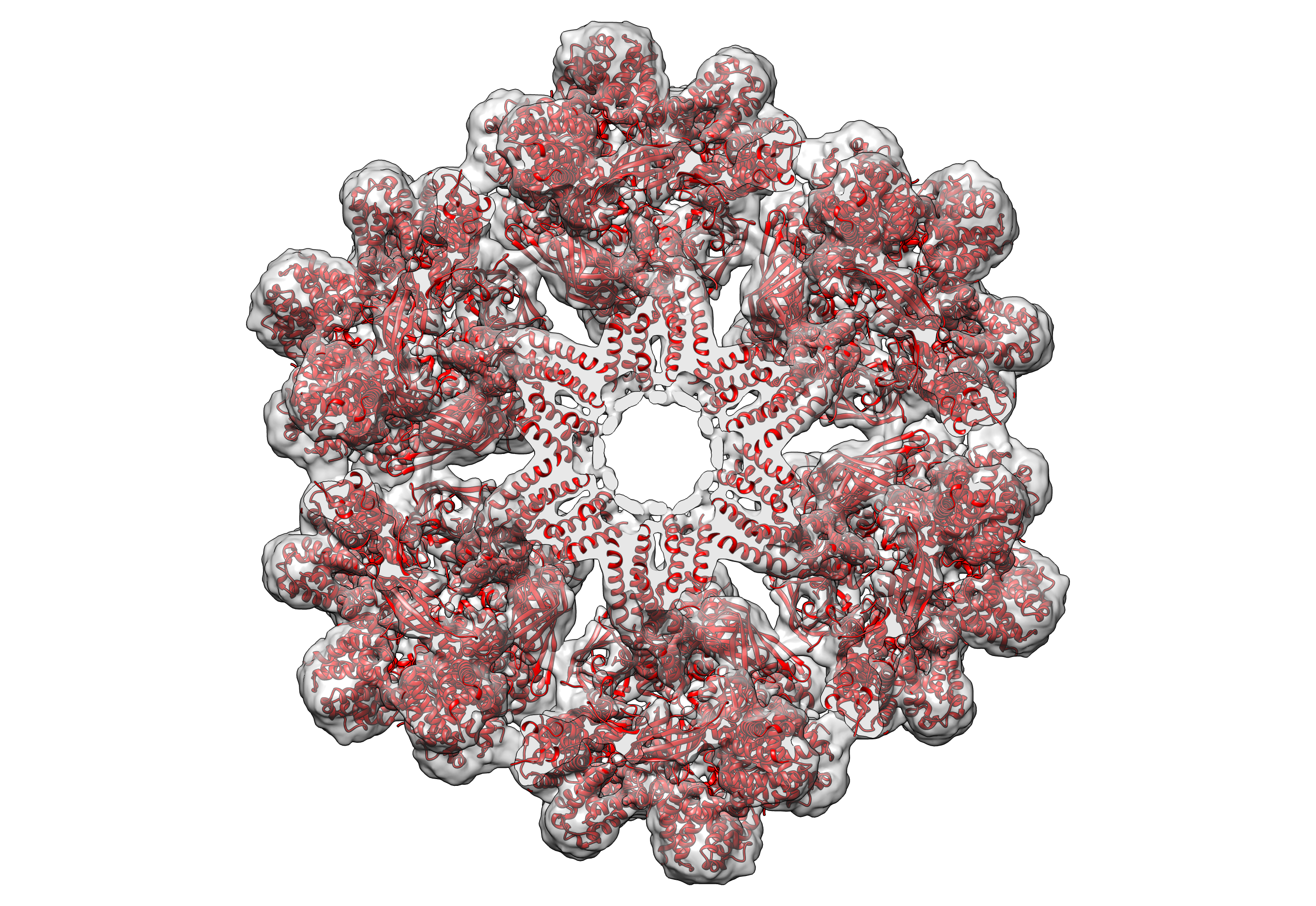


**Supplementary Figure 3. Cut-away view along the six-fold axis with the X-ray model flexibly fitted into the map**. The cutting plane passes through the center of the molecule. The unoccupied central extra density can be discerned easily.
