# Supplementary figures and images for: Structural basis for cooperative oxygen binding and bracelet-assisted assembly of Lumbricus terrestris hemoglobin
Source: Sci Rep. 2015 Apr 21;5:9494. doi: 10.1038/srep09494 (PMC5383013; doi:10.1038/srep09494)

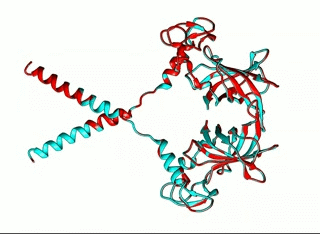

Supplement: Supplementary Information — Supplementary Movie 1 [file srep09494-s2.gif]

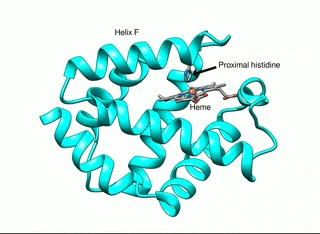

Supplement: Supplementary Information — Supplementary Movie 2 [file srep09494-s3.gif]
